# Polysaccharides from *Dicliptera chinensis* ameliorate liver disturbance by regulating TLR‐4/NF‐κB and AMPK/Nrf2 signalling pathways

**DOI:** 10.1111/jcmm.15286

**Published:** 2020-04-26

**Authors:** Kefeng Zhang, Qiongmei Xu, Ya Gao, Houkang Cao, Yuanyu Lian, Zimeng Li, Jie Xu, Mingli Zhong, Jiani Li, Riming Wei, Jianghui Dong, Ling Jin

**Affiliations:** ^1^ College of Pharmacy Guilin Medical University Guilin China; ^2^ College of Pharmacy Gansu University of Chinese Medicine Lanzhou China

**Keywords:** AMPK/ Nrf2, *Dicliptera chinensis* polysaccharides, liver disturbances, TLR‐4/ NF‐κB

## Abstract

The purpose of this study was to alleviate liver disturbance by applying polysaccharides from *Dicliptera chinensis* (DCP) to act on the adenosine monophosphate–activated protein kinase/ nuclear factor erythroid 2‐related factor 2 (AMPK/ Nrf2) oxidative stress pathway and the Toll‐like receptor 4 (TLR‐4)/ nuclear factor kappa‐B (NF‐κB) inflammatory pathway and to establish an in vivo liver disturbance model using male C57BL/6J and TLR‐4 knockout (^−/−^) mice. For this, we evaluated the expression levels of SREBP‐1 and Nrf2 after silencing the expression of AMPK using siRNA technology. Our results show that with regard to the TLR‐4/ NF‐κB inflammatory pathway, DCP inhibits TLR‐4, up‐regulates the expression of peroxisome proliferator‐activated receptor‐γ (PPAR‐γ), reduces the expression of phospho(p)‐NF‐κB and leads to the reduction of downstream inflammatory factors, such as tumour necrosis factor‐α (TNF‐α), interleukin (IL)‐6 and IL‐1β, thereby inhibiting the inflammatory response. Regarding the AMPK/ Nrf2 oxidative stress pathway, DCP up‐regulates the expression of p‐AMPK and Nrf2, in addition to regulating glucose and lipid metabolism, oxidative stress and ameliorating liver disturbance symptoms. In summary, our study shows that DCP alleviates liver disturbances by inhibiting mechanisms used during liver inflammation and oxidative stress depression, which provides a new strategy for the clinical treatment of liver disturbance.

## 1 INTRODUCTION

Non‐alcoholic fatty liver disease (NAFLD) with an incidence of about 24% of the global adult population is one of the most widespread chronic liver diseases worldwide.^1^ Notably, NAFLD is associated with the development of obesity and type 2 diabetes.^2^ Many studies have found that NAFLD does not only promote end‐stage liver disease, but can also accelerate the occurrence of atherosclerosis, insulin resistance (IR) and tumour development.^2^, ^3^ Importantly, there are no approved drugs specifically registered for the treatment of NAFLD.^4^, ^5^ Based on this, it is clear that identifying novel treatments to improve NAFLD development and treatment is imminent.

The AMPK/ Nrf2 pathway has been shown to play a crucial part in the process of resisting oxidative stress and inflammation in vivo.^6^ Notably, AMPK, a crucial cellular regulator, can maintain the energy supply and demand balance of cells in the body. Activated AMPK promotes activation of Nrf2, which further enhances antioxidant capacity, inhibiting oxidative stress signals.^7^ Studies have also shown that activation of Nrf2 promotes the protein expression of phospho (p)‐Akt, which enhances antioxidant responses and inhibits hepatocyte apoptosis, thus normalizing the liver function of acetaminophen (APAP)‐induced acute liver toxicity in mice.^8^ Hence, the regulation of AMPK/ Nrf2 signals is also a potential strategy for the prevention and treatment of liver disturbance.

The TLR‐4/ NF‐κB signalling pathway acts as an important player in liver inflammation and is closely related to chronic inflammation reactions, such as liver disturbance.^9^, ^10^ Inhibiting TLR‐4/ NF‐κB signals is key in alleviating inflammatory responses in liver tissue, and it is also an indication for down‐regulating inflammatory expression related to oxidative stress.^11^ In addition, the Gardenia fruit glycoside, geniposide, reduces acetaminophen‐induced liver injury in mice, significantly inhibiting inflammatory cell infiltration and reducing the release of inflammatory factors, as well as the protein expression of TLR4 and NF‐κB, which suggests that geniposide‐mediated inhibition via the TLR4/ NF‐κB pathway may help protect hepatocytes.^12^ Thus, inhibiting the TLR‐4/ NF‐κB pathway is a likely candidate target for relieving liver disturbance.

The *Dicliptera chinensis* (L.) Juss (Acanthaceae) polysaccharide (DCP) is a type of natural macromolecular polymer that consists of more than 10 monosaccharides linked by glucoside bonds, used to treat liver diseases due to its plethora of biological activities, including autoxidation, anti‐inflammation and insulin sensitization.^13^ Based on the above studies, we hypothesize that DCP could potentially help alleviate the effects brought on by liver disturbances.

The aim of this study was to investigate the effects and underlying mechanisms mediated by DCP via the AMPK/ Nrf2 pathway by blocking the TLR‐4/ NF‐κB signalling pathway both in vitro and in vivo. This is a crucial step towards exploring novel effective and safe therapies for the treatment of liver disturbances.

## 2 MATERIALS AND METHODS

### 2.1 Ethical approval

All experiments in this study were performed in accordance with the guidelines of the Animal Experimentation Ethics Committee of the Guilin Medical University (approval No. 2019‐0004).

### 2.2 Preparation and characterization of DCP

The flowering plant of *Dicliptera chinensis* (L.) Juss used in the experiment was purchased from the Chinese herbal medicine market. 1 kilogram (Kg) of the plant was chopped and degreased with 95% ethanol at 100 Celsius (℃) in a reflux device for 1.5 hours, and repeated 2 times. The ratio of herb to ethanol was 1:18 (grams [g]/millilitres [mL]). The collected precipitate was dissolved in 1600 mL of water, placed in a dispensing funnel and shaken with butanol/ chloroform (1/5), and then centrifuged to remove the denatured protein. The LGJ‐12 lyophilizer (Beijing Songyuan Huaxing Technology Development Co.) was used to lyophilize the supernatant, and a crude polysaccharide was obtained.

Crude polysaccharides were then dissolved in distilled water, decolorized by activated charcoal and then lyophilized to obtain a refined polysaccharide. The main fraction of DCP had a weight average molecular weight of 2,273 Daltons (Da) and contained glucose, galactose, arabinose, rhamnose and mannose in a 3.20:2.54:1.69:1.58:1.00 molar ratio.

### 2.3 Animal modelling and administration

The Hunan SJA Laboratory Animal (SCXK [Xiang] 2016‐0002, Hunan, China) provided the male C57BL/6J mice used in this study. The Nanjing Biomedical Research Institute of Nanjing University (SCXK [Su] 2015‐0001, Nanjing, China) provided the TLR‐4^−/−^ mice. All mice were fed with standard pellet feed and water under relative humidity (40%‐50%) and temperature (22 ± 2℃). The control day and night period were 12:12 hours, and food and water were available to the mice ad libitum.

For the experimental procedures, 66 male C57BL/6J mice were randomly divided into three groups at the beginning of the experiment, including a Control group (n = 13), a DCP200 group (n = 10) and a Model group (n = 43). The Control and DCP200 groups were fed standard Chow diet, which provided 10% calories from fat, 70% calories from carbohydrate and 20% calories from protein (1 010 001; Jiangsu Xietong Pharmaceutical Bio‐engineering Co.). In contrast, animals in the Model group were fed a high‐fat diet (HFD), which provided 60% calories from fat, 20% calories from carbohydrate and 20% calories from protein (D12492; Jiangsu Xietong Pharmaceutical Bio‐engineering Co.). After 8 weeks of feeding, 3 mice in the Control group and 3 mice in the Model group were sacrificed to evaluate through pathological examination, whether a liver disturbance lesion was formed.^14^ After the liver disturbance model was successfully defined, all mice left in the Model group were further randomly assigned to the HFD group (n = 10) and to the HFD + DCP groups (HFD + DCP50, HFD + DCP100, and HFD + DCP200). The HFD group continued to be fed a HFD for a period of 6 weeks, and the HFD + DCP groups received a combination of a HFD diet and oral DCP for 6 weeks. The HFD + DCP50, HFD + DCP100 and HFD + DCP200 groups were given DCP for intervention at 50, 100 and 200 mg/kg, respectively. At the end of the 14th week, all mice were euthanized to harvest their liver tissues and blood, collected from the abdominal cava vein, for further analysis. In addition, the brain, heart, spleen, lungs and kidneys of the euthanized mice were also fixed with 4% paraformaldehyde.

Moreover, 30 male wild‐type (WT) mice were divided into WT + Chow group, WT + HFD group and WT + HFD + DCP group. Similarly, 30 male TLR‐4^−/−^ mice were randomly divided into TLR‐4^−/−^ + Chow group, TLR −4^−/−^ + HFD group and TLR‐4^−/−^ + HFD + DCP group. The WT + Chow and TLR‐4^−/−^ + Chow group were fed Chow diet, whereas the WT + HFD, WT + HFD +DCP, TLR‐4^−/−^ + HFD and TLR‐4^−/−^ + HFD + DCP groups were fed a HFD for 14 weeks. From the 9th week, DCP (200 mg/kg) was administered to the TLR‐4^−/−^ + HFD + DCP group and the WT + HFD +DCP group for 6 weeks. After 14 weeks, the mice were killed by anaesthesia, and the right lobe of the liver was collected and stored at −80℃. The protein levels of PPAR‐γ, TLR‐4, NF‐κBp65 and p‐NF‐κBp65 in the liver tissue collected from each group were evaluated by Western blot analysis.

### 2.4 Histopathological examination

After 48 hours fixation, the liver tissues were dehydrated and embedded in paraffin. Then, the tissues were cut into sections (4 μm) using the microtome. Sections were then stained according to the haematoxylin‐eosin (H&E) staining methods. Subsequently, sections were treated with xylene and gradient alcohol. After soaking the sections in 95% alcohol, they were then sealed with neutral resin. Moreover, according to the Congo red staining methods (TR‐1302; Beijing Zhongshan Jinqiao Biotechnology Co.), the sections were then stained with Congo red stain, differentiated with alkaline ethanol, counterstained with haematoxylin for 2 minutes and then sealed with neutral resin. Following the above staining procedure, tissue structure, oxidative deposits and lipid deposition were sequentially observed and photographed under a light microscope (Olympus).

### 2.5 Immunohistochemistry

For immunohistochemistry analysis, liver sections were dewaxed and placed in 1x citrate buffer (ThermoFisher Scientific) for high‐pressure antigen repair over 5 minutes. The sections were then allowed to cool naturally, before moving on to blocking procedures. Subsequently, the sections were incubated with a p‐NF‐κBp65 (1:100; Abcam) or an p‐IKKα/β (1:100, Abcam) antibody. The next day, after incubation with respective secondary antibodies, the sections were coloured with the 3, 3'diaminobenzidine (DAB) reagent, red dyed with haematoxylin, dehydrated and dried with ethanol, made transparent with xylene and sealed with neutral gum. These sections were then observed and imaged under the BX51 optical light microscope (Olympus).

### 2.6 in vivo toxicity test

The effect of DCP on liver disturbance remission was observed in our experiments; thus, we further investigated whether a high dose of DCP (eg DCP200) could have an effect on the brain, heart, liver, spleen, lung and kidney tissue structures. Therefore, we collected these organs from the DCP200 group for H&E staining. After sectioning, and fixation procedures, as described above, the sections were stained according to the H&E staining methods. Stained tissue sections were observed and imaged under a light microscope.

### 2.7 Analysis of serum samples

The enzyme activities of alanine aminotransferase (ALT), aspartate aminotransferase (AST), AKP (alkaline phosphatase), gamma‐glutamyl transferase (GGT) and the concentrations of fasting blood glucose (FBG), fasting serum insulin (FINS), free fatty acid (FFA), TC (total cholesterol), TG (total triglyceride), low‐density lipoprotein cholesterol (LDL‐C) and high‐density lipoprotein cholesterol (HDL‐C) were determined in the serum according to instruction manual (Nanjing Jiancheng Bioengineering Institute, Nanjing, China). In addition, the homeostasis model assessment of IR (HOMA‐IR) = serum glucose level × serum insulin level/ 22.5 was determined.^15^ Serum cholesteryl ester transfer protein (CETP) and lecithin cholesterol acyl transferase (LCAT) were measured using an ELISA kit (Wuhan Elabscience Biotechnology Co.).

### 2.8 Analysis of liver tissue samples

Liver tissues were ground into a 10% homogenate (w/v) using phosphate‐buffered saline (PBS) or 0.9% saline for evaluation of oxidative stress and inflammatory response signals. Hepatic contents of TG, superoxide dismutase (SOD), malondialdehyde (MDA) and glutathione peroxidase (GSH‐Px) were detected according to the manufacturer's procedure (Nanjing Jiancheng Bioengineering Institute).

The hepatic TNF‐α, IL‐1β and IL‐6 were measured using the ELISA kit following the manufacturer's instructions provided (Wuhan Elabscience Biotechnology Co.). A microplate reader (Bio Tek, Winooski, VT, USA) was used to detect the absorbance at 450 nm. The TNF‐α, IL‐1β and IL‐6 detected in liver tissue were calculated according to a standard curve.

### 2.9 siRNA‐mediated protein down‐regulation

HepG2 cells (ATCC, Rockefeller, MA, USA) were cultured in Dulbecco's modified Eagle's medium (DMEM; Gibco, USA) supplemented with 10% foetal bovine serum (900‐108; Gemini, CA, USA), 100 U/mL of penicillin and 100 μg/mL of streptomycin in 37℃, 5% CO_2_ incubators. HepG2 cells were then divided into 5 groups: Control, FFA (1 mM),^16^ siAMPK, siAMPK + FFA and siAMPK + FFA + DCP groups. Double‐stranded small‐interfering RNA (siRNA) technology targeting human AMPK mRNA was used to silence AMPK expression in HepG2 cells. In addition, a control siRNA was used to test for possible non‐specific effects of siRNA. Finally, HepG2 cells were transfected with siRNA using the lipid agent, Lipofectamine 2000 (Invitrogen) reagent and incubated for 36 hours.

### 2.10 Real‐time reverse transcription polymerase chain reaction

A TRIzol reagent was used to extract the total RNA of liver tissues (Beyotime Biotechnology Co.). Next, according to the real‐time reverse transcription polymerase chain reaction (RT‐PCR) kit instructions (Beijing ComWin Biotech Co.), and based on the RNA template, the reverse transcriptase enzyme provided was used to reversely transcribe the total RNA into complementary DNA (cDNA). The cDNA was amplified on a CFX96 fluorescence quantitative PCR instrument (Bio‐Rad, Hercules, CA, USA) according to the reaction conditions of the detection kit (Cwbio Century Biotechnology Co.). In brief, samples were treated under the conditions of 95℃ for 10 minutes, 95℃ for 15 seconds (s) and 60℃ for 1 minutes, and the cycle was performed 40 times. Expressions of target genes were carried out by a comparative method (2^−ΔΔCt^), with β‐actin used as an internal reference. The primer sequences utilized are shown in Table 1 (Huada Gene Research Institute, Shenzhen, China).

**Table 1:** Primer sequences

Genes	Primer	Sequence (5'‐3')	Tm (℃)	Product length	GenBank code
PPAR‐γ	Forward	TCGCTGATGCACTGCCTATG	60.53	103	19 016
	Reverse	GAGAGGTCCACAGAGCTGATT	59.17		
NF‐κB	Forward	CCCTGAGAAAGAAACACAAGGT	58.44	289	18 033
	Reverse	ATGAAGGTGGATGATGGCTAAG	57.9		
TLR‐4	Forward	AGGATGATGCCAGGATGATGTC	59.96	320	21 898
	Reverse	TCAGGTCCAGGTTCTTGGTTGAG	62.08		
SREBP‐1	Forward	CCATTGACAAGGCCATGC	56.76	162	20 787
	Reverse	GGTCATGTTGGAAACCACGC	60.04		
AMPK	Forward	GTCAAAGCCGACCCAATGATA	58.36	100	105 787
	Reverse	CGTACACGCAAATAATAGGGGTT	59.13		
ACC1	Forward	TCACACCTGAAGACCTTAAAGCC	60.5	152	107 476
	Reverse	AGCCCACACTGCTTGTACTG	60.25		
FAS	Forward	TCTGGTTCTTACGTCTGTTGC	58.25	197	14 102
	Reverse	CTGTGCAGTCCCTAGCTTTCC	60.68		
SCD1	Forward	TTCCTACCTGCAAGTTCTACACC	59.99	116	20 249
	Reverse	CCGAGCTTTGTAAGAGCGGT	60.39		
β‐actin	Forward	GGAGATTACTGCCCTGGCTCCTA	63.18	150	11 461
	Reverse	GACTCATCGTACTCCTGCTTGCTG	63.25		

### 2.11 Western blot analysis

For protein analysis, 50 mg of liver tissue samples was lysed using the radioimmunoprecipitation assay (RIPA) buffer containing 1% (v/v) phenylmethylsulphonyl fluoride (PMSF, P0013B; Beyotime Biotechnology Co.). After the protein supernatant was collected, the bicinchoninic acid (BCA) protein assay kit (P0010S; Beyotime Biotechnology Co.) was utilized to detect the protein content of the supernatant. Next, a protein loading buffer was added and protein denaturation was carried out in a 95℃ water bath for 5 minutes. Proteins were then separated using sodium dodecyl sulphate‐polyacrylamide gel electrophoresis (SDS‐PAGE). Then, the proteins were shifted onto a polyvinylidene fluoride (PVDF) membrane (Millipore). The PVDF membranes were then blocked in Western blocking buffer at room temperature and incubated overnight at 4℃ using the following primary antibodies: β‐actin (1:2000, Proteintech), AMPK, p‐AMPK, NF‐κBp65 (1:1000, Cell Signaling Technology), sterol regulatory element‐binding protein‐1(SREBP‐1), PPAR‐γ, TLR4 and p‐NF‐κBp65 (1:1000, Abcam). The next day, the membranes were washed twice with tris‐buffered saline, 0.1% Tween 20 (TBST) buffer, and then incubated for 2 hours with their respective secondary antibodies (1:2000, Proteintech) in Western secondary antibody dilution buffer. All blot intensities were quantified using the Tanon 4600 fully automated chemiluminescence image analysis system (Tanon Technology Co.). Protein expressions were quantified using Image‐Pro Plus version 6.0 software (Media Cybernetics) normalized against the housekeeping protein β‐actin.

### 2.12 Statistical analysis

The GraphPad Prism 5.0 software (GraphPad) was used to analyse the data, which was expressed as the mean ± standard deviation (SD). To assess the differences between groups, one‐way analysis of variance was used. The independent sample* t* test was used for comparison between the two groups. A *P* value of less than (<) .05 indicated statistical significance.

## 3 RESULTS

### 3.1 Establishment of the liver disturbance model

**Figure 1:**
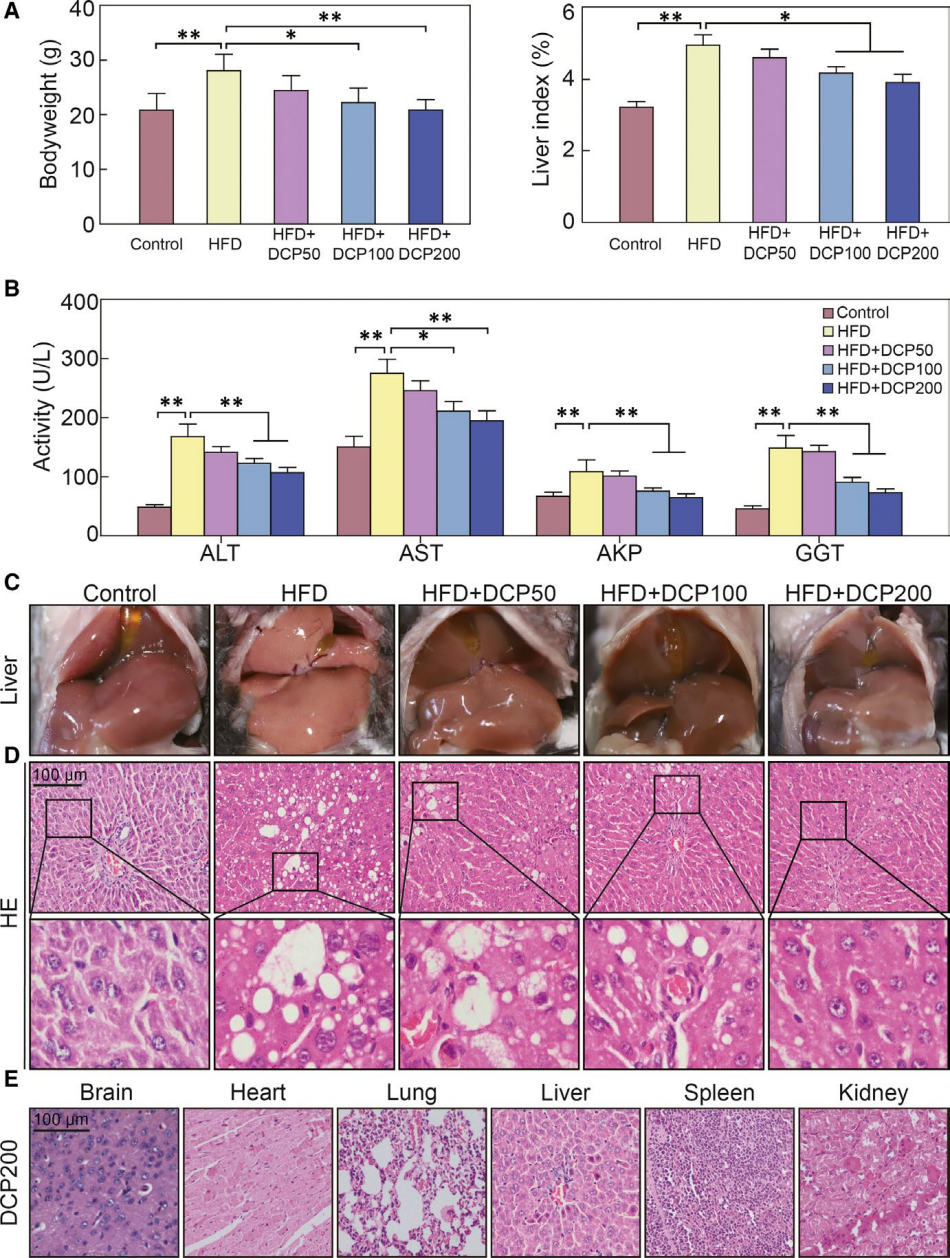
Effect of DCP on liver function. A, Bodyweights and liver indexes of treated mice. B, ALT, AST, AKP and GGT activity levels in collected serum samples. C, Liver morphology and (D) H&E staining. E, H&E staining of the brain, heart, liver, spleen, lungs and kidneys from the DCP200 group. All data are presented as the means ± standard deviation [SD] (n = 10; *P < .05, **P < .01). Bar = 100 μm

Compared to the Control group, the body weights and liver indexes of HFD group were markedly increased (Figure 1A). Moreover, the HFD group exhibited significant enhancement in serum liver enzyme activity, including ALT, AST, AKP and GGT（Figure 1B). In the Control group, the liver surface was bright red and smooth and exhibited integrated cords of hepatocytes which radiated from the central vein and presented a normal hepatic lobular structure (Figure 1C,D). In the HFD group, liver tissue samples presented a yellowish colour and had increased in volume. In addition, liver sections displayed macro‐ and microvascular steatosis, slight inflammatory cell infiltration and ballooning degeneration (Figure 1C,D). These data show that we successfully established a liver disturbance model.

### 3.2 The effect of DCP on liver function

Compared to the HFD group, there are significant decreases in the body weights and liver indexes of the HFD + DCP200 group (Figure 1A). Moreover, the enzyme activity of ALT, AST, AKP and GGT were markedly reduced (Figure 1B). Liver tissues of the HFD + DCP group presented a liver colour that was bright red and shiny (Figure 1C), with the pathological lesions ameliorating distinctly and a lowered accumulation of fat vacuoles and necrotic cells (Figure 1D). H&E analysis demonstrated that no significant pathological differences (Figure 1E), indicating that DCP had no obvious toxic side effects to the other main organs in HFD + DCP group.

### 3.3 DCP effects on lipid accumulation

**Figure 2:**
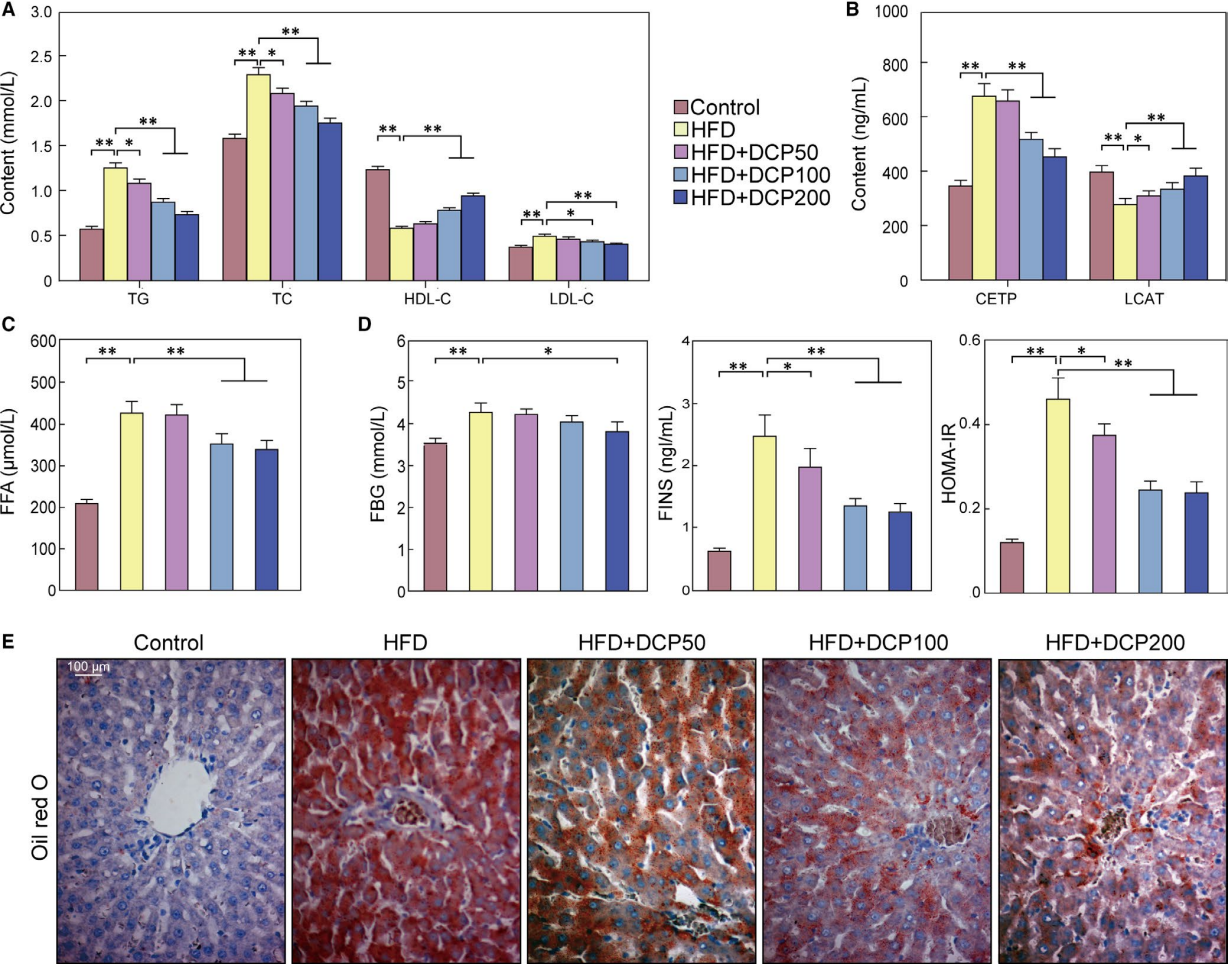
Effect of DCP on liver lipid accumulation. A, TG, TC, HDL‐C and LDL‐C serum content levels. B, CETP and LCAT levels. C, FFA serum levels. D, FBG, FINS and HOMA‐IR content analysis. E, Liver Oil red O staining histology. All data are presented as the means ± SD (n = 10; *P < .05, **P < .01). Bar = 100 μm

Serum lipid profiles, including TC, TG, LDL‐C, CETP and FFA, were dramatically elevated in the HFD group, whereas the serum content of HDL‐C and LCAT decreased markedly, when compared to the Control group (Figure 2A‐C). In addition, the FBG, FINS and HOMA‐IR levels were markedly increased in the HFD group relative to the Control group (Figure 2D). Notably, in the HFD group, Oil Red O staining showed a large area of red lipid drops with obvious inflammatory cell infiltration (Figure 2E). These data demonstrate that lipid accumulation is significant in our liver disturbance model and that DCP treatment has beneficial effects on liver and serum lipid levels.

### 3.4 DCP effect on liver oxidative stress signals

**Figure 3:**
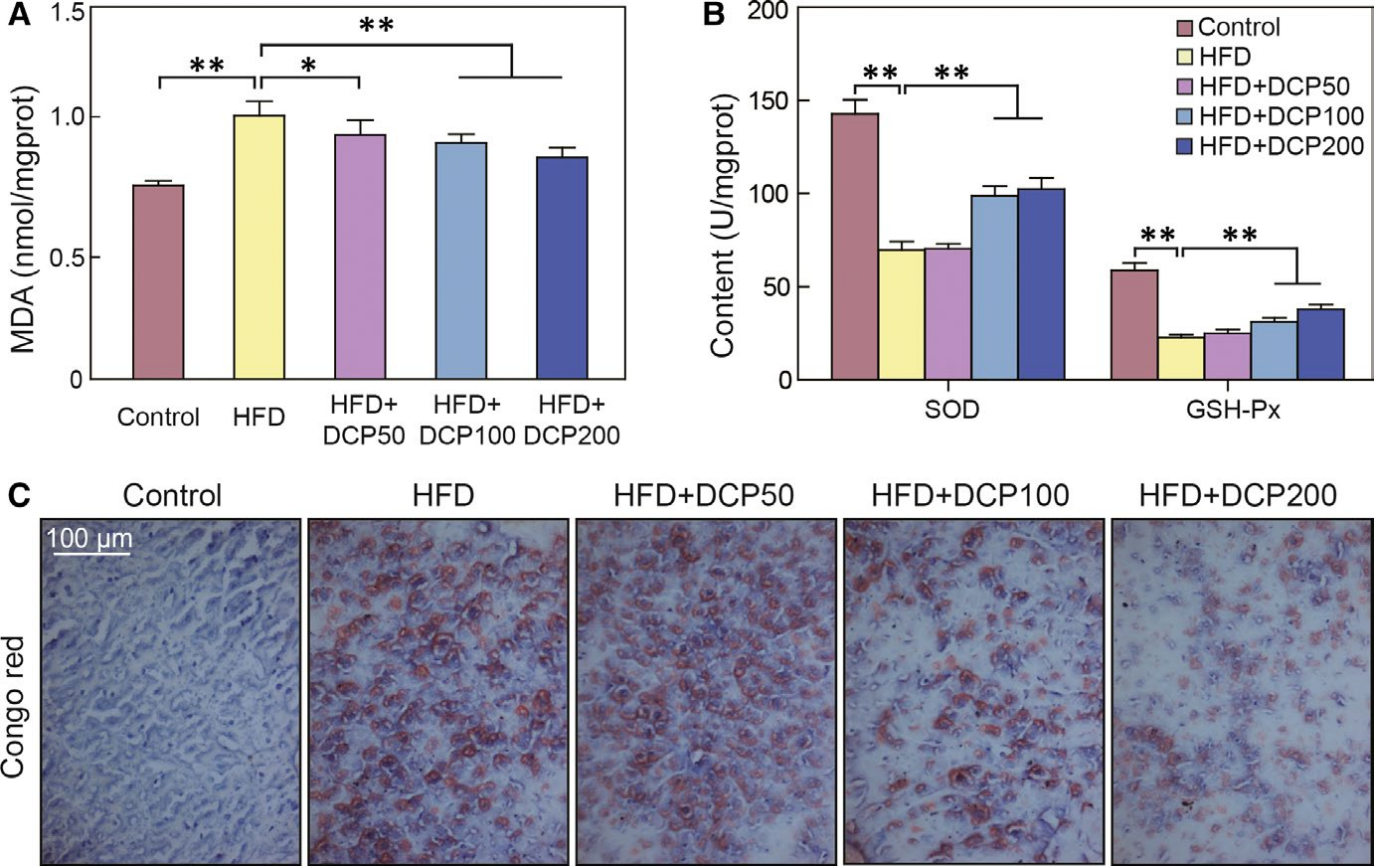
The effect of DCP on oxidative stress pathways in the liver. A, MDA liver content. B, SOD and GSH‐Px levels. C, Congo red stain analysis. All data are presented as the means ± SD (n = 10; *P < .05, **P < .01). Bar = 100 μm

When compared to those of the HFD group, the enzyme activity of SOD and GSH‐Px were markedly increased and the MDA content in the liver was reduced dramatically in the HFD + DCP200 group (Figure 3A,B). Notably, Congo red staining analysis in the liver of the HFD group showed a large amount of red sediment accumulation. This accumulation was significantly reduced in the HFD + DCP200 group (Figure 3C). These data show that DCP can significantly inhibit oxidative stress signalling pathways.

### 3.5 The effect of DCP on the AMPK/ Nrf2 liver pathways

**Figure 4:**
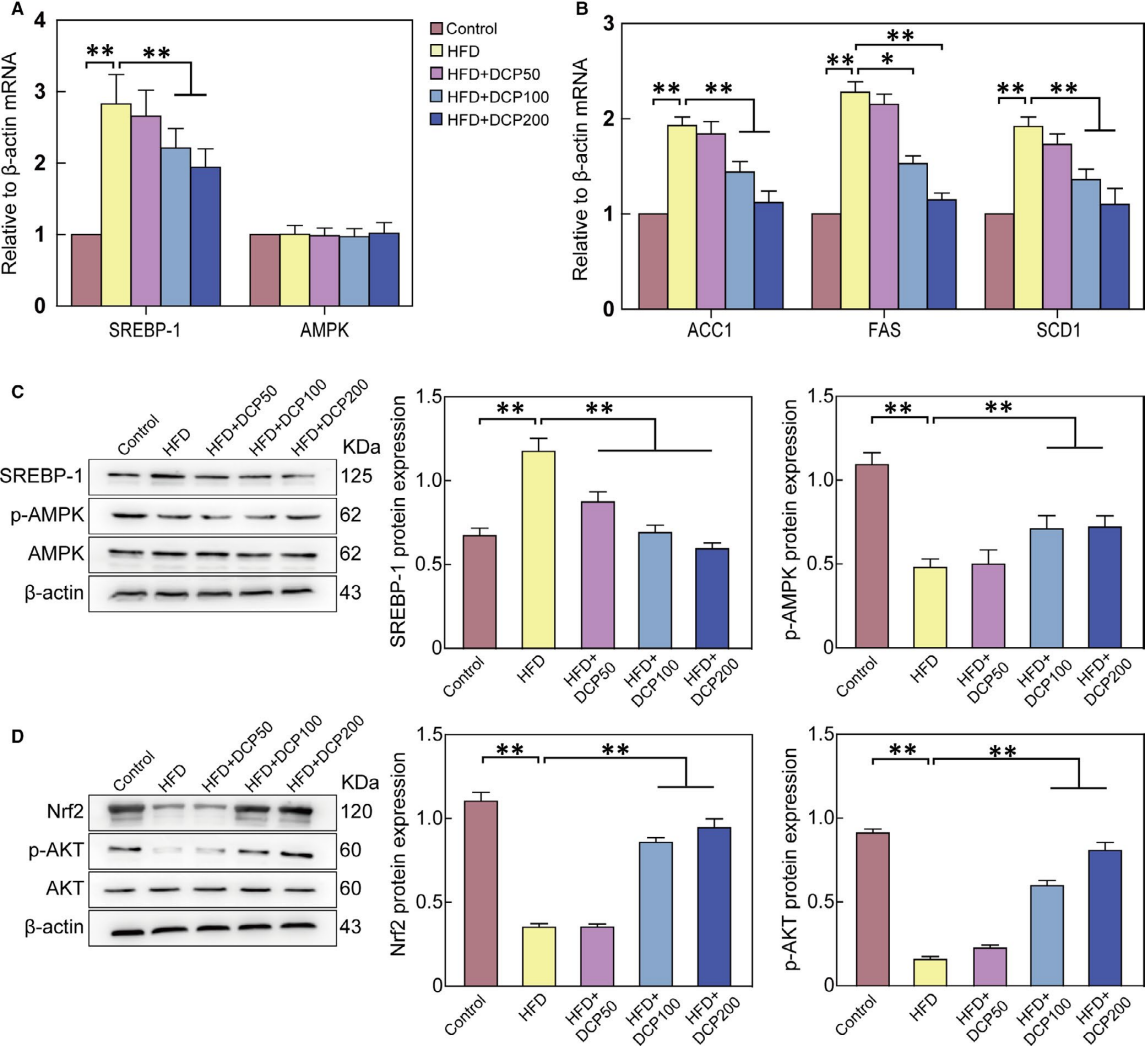
The effect of DCP on the AMPK/ Nrf2 liver signalling pathway. A, mRNA expression levels of SREBP‐1 and AMPK. B, mRNA expression levels of ACC1, FAS and SCD1. C, Hepatic protein expression of SREBP‐1, p‐AMPK, and AMPK. D, Hepatic protein expression of Nrf2, p‐AKT and AKT. All data are presented as the means ± SD (n = 3; *P < .05, **P < .01)

Notably, the HFD group showed no significant changes in AMPK mRNA levels; however, SREBP‐1, acetyl‐CoA carboxylase 1 (ACC1), fatty acid synthase (FAS), stearoyl‐coenzyme A desaturase 1 (SCD1) mRNA were significantly increased, when compared to the Control group, (Figure 4A,B). In contrast, p‐AMPK protein expression levels decreased and SREBP‐1 protein expression levels increased in the HFD group (Figure 4C). In addition, the expression levels of p‐AKT and Nrf2 proteins in the HFD group were lower than those of the Control group (Figure 4D).

### 3.6 Effects of DCP on HepG2 AMPK/ Nrf2 signalling pathways

**Figure 5:**
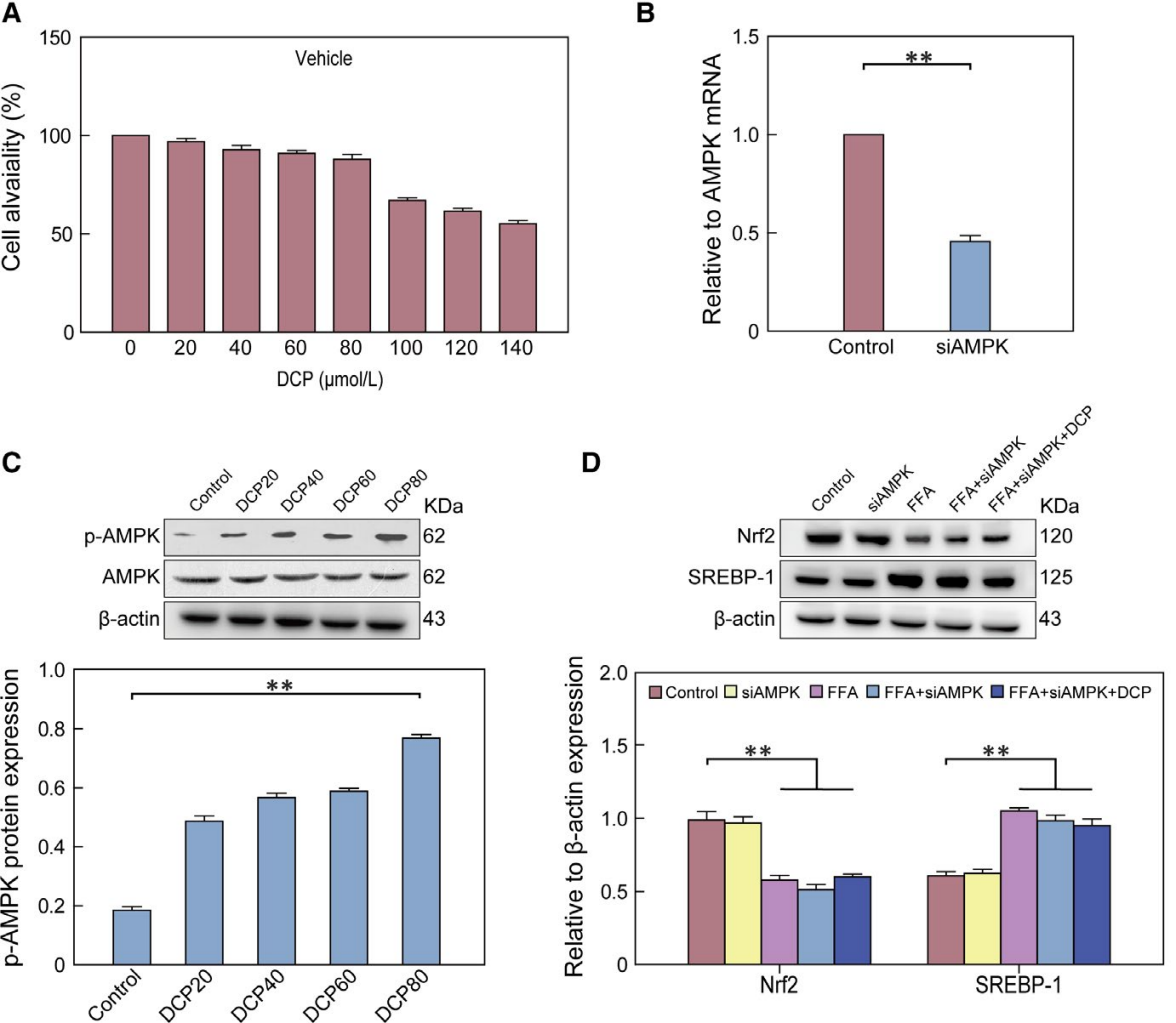
The effect of DCP on AMPK/ Nrf2 signalling pathways in HepG2 cells. A, Evaluating a safe DCP concentration for dose administration. B, mRNA expression levels of AMPK. C, Western blot analysis showing the protein expression of p‐AMPK and AMPK. D, Nrf2 and SREBP‐1 protein levels. All data are presented as the means ± SD (n = 3; *P < .05, **P < .01)

At a concentration of 80 μmol/L of DCP, we observed stable cell viability and high levels of p‐AMPK. Therefore, we chose this concentration as the effective concentration for our studies (Figure 5A,C). Compared with the Control group, the expression levels of SREBP‐1 and Nrf2 in the siAMPK group were not statistically significant (Figure 5D). After silencing the expression of AMPK using siRNA, SREBP‐1expression increased, whereas the expression of Nrf2 decreased in the siAMPK + FFA group, when compared to the FFA group (Figure 5B).

After DCP treatment, we found no statistically significant differences in the expression levels of SREBP‐1 and Nrf2 between the DCP + siAMPK + FFA group and the siAMPK + FFA group (Figure 5D). These results demonstrate that DCP can improve liver disturbance by activating AMPK/ Nrf2 signalling pathways.

### 3.7 The effect of DCP on TLR‐4/ NF‐κB liver pathways

**Figure 6:**
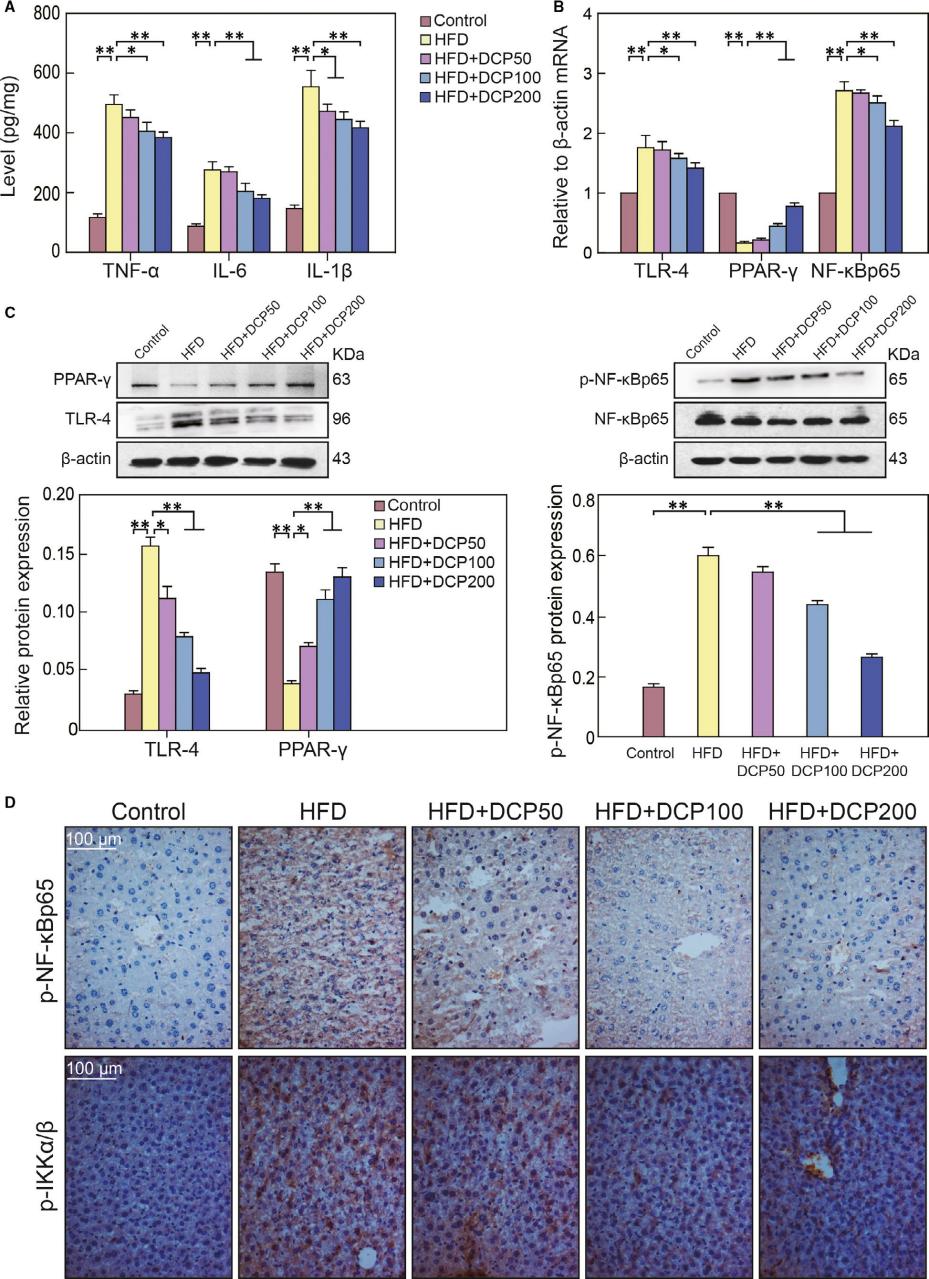
The effect of DCP on TLR‐4/ NF‐κB signals. A, TNF‐α, IL‐6 and IL‐1β liver expression levels. B, TLR‐4, PPAR‐γ and NF‐κBp65 mRNA expression profiles. C, Hepatic protein expression of PPAR‐γ, TLR‐4, p‐NF‐κBp65 and NF‐κBp65. D, Immunohistochemical analysis of p‐NF‐κBp65 and p‐IKKα/β expression levels. All data are presented as the mean ± SD (n = 3; *P < .05, **P < .01). Bar = 100 μm

When compared to the Control group, the hepatic levels of TNF‐α, IL‐1β and IL‐6 in the HFD group were significantly elevated when reflecting the inflammatory response typical of liver disturbance (Figure 6A). Furthermore, RT‐PCR analysis showed that PPAR‐γ mRNA levels were remarkedly decreased in the HFD group, whereas TLR‐4 and NF‐κBp65 mRNA levels were significantly elevated (Figure 6B). The expression of PPAR‐γ proteins in the HFD group was down‐regulated whereas TLR‐4 and p‐NF‐κBp65 levels were significantly increased (Figure 6C). Immunohistochemical analysis also showed that the levels of p‐NF‐κBp65 and p‐IKKα/β in the liver tissue collected from HFD were particularly increased (Figure 6D). These data indicate that the inflammatory response mediated by liver disturbance is closely connected with the TLR‐4/ NF‐κB signalling pathway.

### 3.8 Effects of DCP on TLR‐4/ NF‐κB signals of TLR‐4^−/−^mice

**Figure 7:**
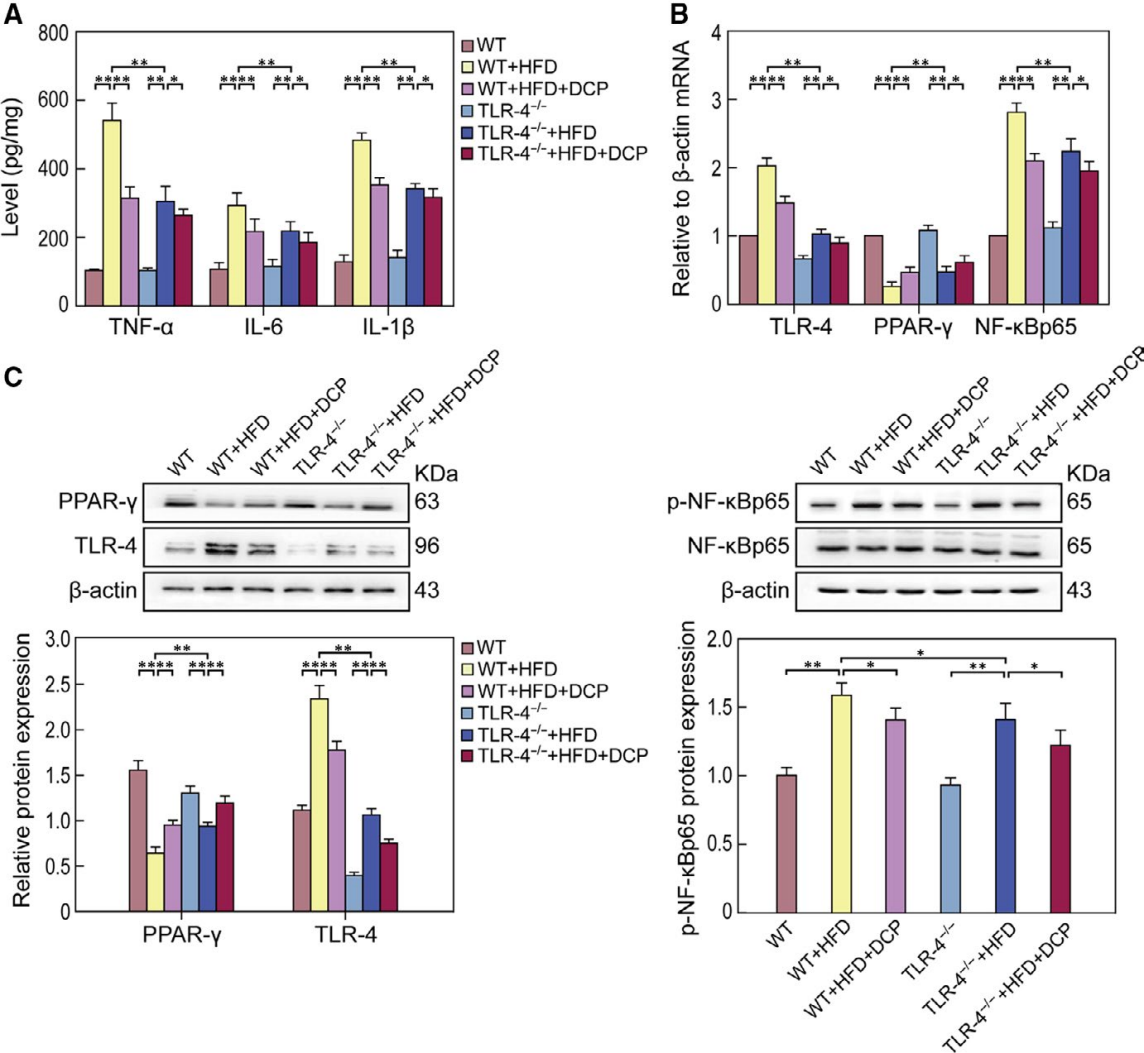
Effect of DCP on TLR‐4/ NF‐κB signals of TLR‐4^−/−^ mice. A, Levels of TNF‐α, IL‐6 and IL‐1β in the liver. B, Expression profiles of liver TLR‐4, PPAR‐γ and NF‐κBp65 mRNA levels. C, Hepatic protein expression of PPAR‐γ, TLR‐4, p‐NF‐κBp65 and NF‐κBp65. All data are presented as the mean ± SD (n = 3; *P < .05, **P < .01)

The mRNA hepatic levels of TNF‐α, IL‐1β, IL‐6, TLR‐4 and NF‐κBp65 in the WT + HFD and TLR‐4^−/−^ + HFD groups were significantly higher than those of the WT and TLR‐4^−/−^ groups, whereas PPAR‐γ mRNA levels were lower (Figure 7A,B). Moreover, the protein expression levels of PPAR‐γ in the TLR‐4^−/−^ + HFD group was higher than that in the WT + HFD group, and the protein levels of TLR‐4 and NF‐κBp65 were lower than in the TLR‐4^−/−^ + HFD group (Figure 7C).

When compared to the TLR‐4^−/−^ + HFD group, the mRNA levels of TNF‐α, IL‐6, IL‐1β, TLR‐4 and NF‐κBp65 were lower, and PPAR‐γ levels were higher, whereas protein expression levels of PPAR‐γ were higher, and TLR‐4 and NF‐κBp65 were lower in the TLR‐4^−/−^ + HFD + DCP group (Figure 7). These results show that DCP can inhibit the TLR‐4/ NF‐κB pathway and ameliorate liver disturbance symptoms.

## 4 DISCUSSION

The AMPK/ Nrf2 signalling pathway can regulate lipid metabolism in liver disturbance.^17^ Meanwhile, chronic inflammatory responses such as the ones associated with liver disturbance have been shown to be closely connected with the TLR‐4/ NF‐κB signalling pathway.^12^ In this study, C57BL/6J mice were fed a HFD to establish a liver disturbance model and evaluate the effect of DCP on liver function, glycolipid metabolism, oxidative stress and inflammation. For this, TLR‐4^−/−^ mice were used in order to validate the in vivo effects of DCP on the TLR‐4/ NF‐κB pathways. The results revealed that DCP can regulate the mRNA and protein expression levels of TLR4/ NF‐κB signalling pathway components and also inhibit fatty acid synthesis in the liver by interfering with the AMPK/ Nrf2 signalling pathway. In summary, DCP can block liver inflammation, and also regulate lipid metabolism, thereby reducing liver disturbance symptoms.

In the current study, we successfully established a liver disturbance model using an enriched diet. Notably, this diet is known to increase FFA in blood serum. The continuous accumulation of FFA in the body causes liver damage and can lead to the overflow of ALT and AST enzymes into the blood.^18^ In addition, an HFD diet has been shown to elevate ALT, AST, AKP and/ or GGT enzyme activities in the serum of an liver disturbance C57BL/6J mouse model.^19^ Here, the serum enzyme activities of ALT, AST, AKP and GGT of the HFD group were noticeably increased after administration of a HFD (Figure 1A). The serum contents of TG, TC and LDL‐C of the HFD mouse group increased significantly, whereas the content of HDL‐C was significantly reduced (Figure 2A). Markedly, HFD‐induced liver disturbance in mice showed a presence of IR and lipid disorders, and increased FFA content (Figure 2).

In this study, DCP inhibited AMPK/ SREBP‐1 signalling pathways, regulated glucose and lipid metabolism, and inhibited IR, thereby improving liver disturbance symptoms and reducing its progression. For instance, after DCP was given to C57BL/6J mice, protein expression levels of p‐AMPK increased, and SREBP‐1 levels decreased (Figure 4). Markedly, trapaquadrispinosa polyphenol‐rich pericarp extract (caltrop) has been shown to be able to inhibit liver disturbance by regulating the expression of p‐AMPK in the AMPK/ SREBP‐1 pathway, whereas reducing the expression of the SREBP‐1 protein.^20^ In addition, the oil, via Allyl isothiocyanate, can also up‐regulate liver protein levels of p‐AMPK in C57B/6 mice, thus relieving lipid accumulation.^21^ Lipid metabolism, oxidative stress and inflammatory responses are key aspects of the pathological processes of liver disturbances.^22^ Notably, the AMPK can phosphorylate the 372 sites of SREBP‐1, inhibit SREBP‐1 cleavage and nuclear translocation, and reduce TG and lipid accumulation in liver cells. In addition, elevated TC and TG levels can lead to IR, which can in turn inhibit AMPK activity, thus leading to a vicious and detrimental cycle in the body.^23^ IR promotes lipid production and fatty acid flux to the liver, and in certain cases can aggravate liver steatosis.^24^ SREBP‐1c transgenic mice present obvious obstacles to adipocyte differentiation leading to faster FFA synthesis and increased liver TG accumulation,^25^ whereas SREBP‐1c activates the transcription process of lipid synthesis by regulating ACC1, FAS and SCD1.^26^

In this study, DCP was found to be able to regulate protein expression levels in lipid transport system components, which in turn improves lipid metabolism. In addition, DCP reduced CETP levels and increased LCAT levels in the serum of mice under HFD conditions (Figure 2B). siAMPK transfection experiments also showed the regulatory effects of DCP on AMPK/ Nrf2 pathways, as they relate to AMPK signals (Figure 5). Abnormal lipid transport is also an important factor for lipid accumulation in liver cells. Excessive intake of exogenous cholesterol can enhance the expression of CETP, inhibiting LCAT levels, which can cause the reverse transport of cholesterol from surrounding tissues into the liver, breaking the balance of lipid metabolism in the body.^27^ Moreover, the activation of AMPK can lead to the activation of Nrf2, which further promotes the transcription and expression of downstream antioxidant enzyme genes, enhancing the innate antioxidant capacity of cells, inhibiting oxidative stress and slowing cell damage.^28^, ^29^

In addition, DCP also inhibited the TLR‐4/ NF‐κB pathway, reduced inflammatory responses. It inhibited the release of TNF‐α, IL‐6 and IL‐1β, reducing the inflammatory response and protecting liver function (Figure 6A). Moreover, it significantly up‐regulated PPAR‐γ expression, down‐regulated TLR‐4 expression and inhibited p‐NF‐κBp65 levels (Figure 6B‐C). Therefore, DCP plays a regulatory role in the TLR‐4/ NF‐κB signalling pathway and inhibits TNF‐α, IL‐6 and IL‐1β levels to alleviate liver disturbance. Nrf2, NF‐ κB and PPAR‐γ are key nuclear transcription factors that play important roles in the regulation of liver inflammation.^30^ Interestingly, the TLR‐4 receptor can bind to its unique ligand to activate NF‐κB, thereby releasing TNF‐α, IL‐6 and IL‐1β in the circulation. On the other hand, a HFD diet can cause an increase in fatty acids, which in turn can lead to the increase of inflammation‐related factors such as NF‐kB a and TNF‐α.^31^ Decreased gene expression and PPAR transcription factor family activities are closely related to an increase of the nuclear transcription factor activity of NF‐kB.^31^, ^32^, ^33^

In order to further investigate the target of DCP inhibition on the TLR‐4/ NF‐κB pathway, TLR‐4^−/−^ mice under a HFD were fed as an in vivo liver disturbance model. mRNA and protein expression levels of PPAR‐γ were up‐regulated in the WT + HFD + DCP group, whereas the expression levels of TLR‐4 and p‐NF‐κBp65 were significantly down‐regulated (Figure 7). This is consistent with the related proteins, which participate in the TLR‐4/ NF‐κB pathway in the TLR‐4^−/−^ + HFD mice. Our data indicate that the inflammatory response in liver disturbance is closely connected with TLR‐4/ NF‐κB signals. Astragalus polysaccharides and TLR4‐deficient mice have decreased levels of TNF‐α, IL‐1β and IL‐6 and decreased protein expression of p‐NF‐κBp65.^34^ DCP inhibits the protein expression of TLR‐4 and p‐NF‐κBp65 as effectively as the levels observed in the TLR‐4^−/−^mice. It thus can alleviate liver disturbance inhibiting liver inflammatory response and oxidative stress.

**Figure 8:**
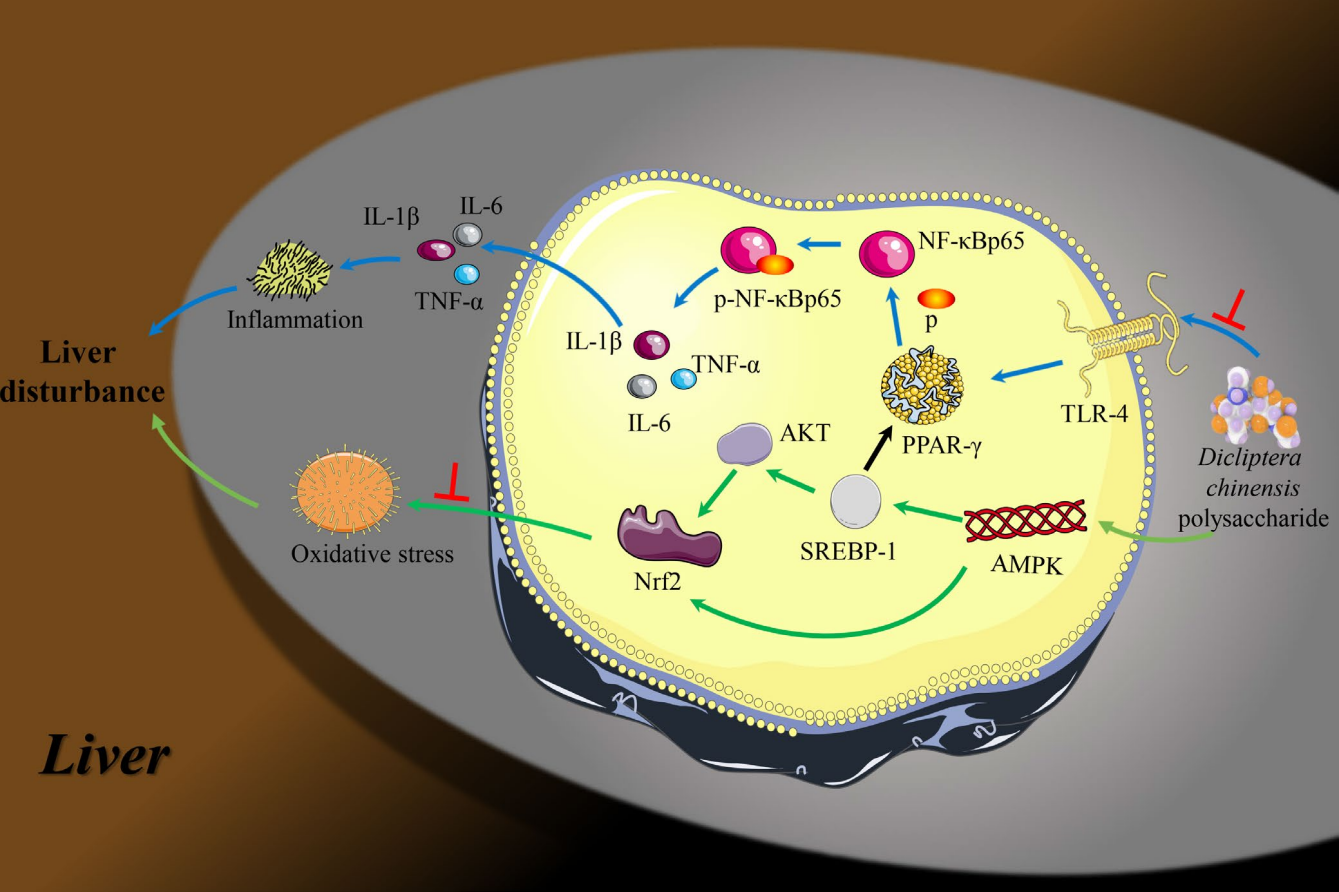
Proposed model depicting the underlying mechanisms of DCP in regulating liver inflammation responses and oxidative stress in liver disturbance

In summary, DCP is the potential new treatment for the prevention and treatment of liver disturbance. It can reduce the progression of liver disturbance by inhibiting the AMPK/ Nrf2 pathway and blocking the TLR‐4/ NF‐κB signalling pathway (Figure 8). DCP can up‐regulate the protein expression of p‐AMPK, p‐AKT and Nrf2, reduce the protein expression of SREBP‐1, regulate glucose and lipid metabolism and oxidative stress, and improve liver disturbance symptoms. On the other hand, DCP can inhibit TLR‐4, up‐regulate the expression of PPAR‐γ and reduce the expression of p‐NF‐κB, which can lead to the reduction of downstream inflammatory factors involving TNF‐α, IL‐6 and IL‐1β signals. Importantly, inflammatory responses are, as a result, significantly reduced. These results provide a new strategy for DCP, as a treatment option in the clinical management of liver disturbance.

## CONFLICT OF INTEREST

None.

## AUTHOR CONTRIBUTIONS

KZ, JD and LJ designed the study; QX, YG, YL, MZ and JL performed experiments; ZL, JX, RW and drew the figure and the table; KZ, QX, HC, JD and LJ wrote the manuscript. All authors read and approved the final manuscript.

## Data Availability

All data included in this study are available upon request by contact with the corresponding author.
